# Vaccines and Therapeutics Against Hantaviruses

**DOI:** 10.3389/fmicb.2019.02989

**Published:** 2020-01-30

**Authors:** Rongrong Liu, Hongwei Ma, Jiayi Shu, Qiang Zhang, Mingwei Han, Ziyu Liu, Xia Jin, Fanglin Zhang, Xingan Wu

**Affiliations:** ^1^Department of Microbiology, School of Basic Medicine, Fourth Military Medical University, Xi’an, China; ^2^Scientific Research Center, Shanghai Public Health Clinical Center & Institutes of Biomedical Sciences, Key Laboratory of Medical Molecular Virology of Ministry of Education & Health, Shanghai Medical College, Fudan University, Shanghai, China; ^3^Viral Disease and Vaccine Translational Research Unit, Institut Pasteur of Shanghai, Chinese Academy of Sciences, Shanghai, China; ^4^School of Biology and Basic Medical Sciences, Soochow University, Suzhou, China; ^5^Cadet Brigade, School of Basic Medicine, Fourth Military Medical University, Xi’an, China

**Keywords:** hantavirus, vaccine, therapeutic strategies, HFRS, HPCS

## Abstract

Hantaviruses (HVs) are rodent-transmitted viruses that can cause hantavirus cardiopulmonary syndrome (HCPS) in the Americas and hemorrhagic fever with renal syndrome (HFRS) in Eurasia. Together, these viruses have annually caused approximately 200,000 human infections worldwide in recent years, with a case fatality rate of 5–15% for HFRS and up to 40% for HCPS. There is currently no effective treatment available for either HFRS or HCPS. Only whole virus inactivated vaccines against HTNV or SEOV are licensed for use in the Republic of Korea and China, but the protective efficacies of these vaccines are uncertain. To a large extent, the immune correlates of protection against hantavirus are not known. In this review, we summarized the epidemiology, virology, and pathogenesis of four HFRS-causing viruses, HTNV, SEOV, PUUV, and DOBV, and two HCPS-causing viruses, ANDV and SNV, and then discussed the existing knowledge on vaccines and therapeutics against these diseases. We think that this information will shed light on the rational development of new vaccines and treatments.

## Introduction

In recent years, the repeated outbreak of hantavirus disease has caused a serious threat to human health. The spread of hantavirus from natural hosts to humans is a natural ecological process; however, the outbreak of hantavirus is driven by striped field mouse population cycle dynamics and seasonal climate change (Tian and Stenseth, 2019).

Hantavirus is a virus transmitted mainly by rodent animals, mainly through urine, feces, and saliva and the aerosols produced by them, but rarely by the bites of infected animals (Brocato and Hooper, 2019). In recent years, the infection rate of hantavirus has increased in China and Europe (Dong et al., 2019). Hantavirus disease has turned out to be a newly identified but not a “new” disease in Germany (Kruger et al., 2013). The clinical presentations may vary according to viral strains prevalence in different regions. In Asia, hantavirus infection by Hantan virus (HTNV) and Seoul virus (SEOV) targets mainly the human kidney and causes hemorrhagic fever with renal syndrome (HFRS). In North America, infection by Andes virus (ANDV) and Sin Nombre virus (SNV) manifests in mainly the lung and leads to hantavirus pulmonary syndrome (HPS) or hantavirus cardiopulmonary syndrome (HCPS), with high mortality rates; in Europe, infection by Puumala virus (PUUV) and Dobrava-Belgrade virus (DOBV) typically causes a milder form of HFRS, nephropathia epidemica (NE) (Echterdiek et al., 2019).

Currently, there is no approved post-exposure therapeutic countermeasure against hantaviral infection, but diversified treatment strategies have been developed and applied to manage HFRS or HCPS. These strategies target viral life cycle, host immunological factors, or patient clinical symptoms. Preventive measures against hantaviral infection, especially vaccine development, are essential for future pandemics. In this paper, we reviewed the epidemiology and pathogenesis of hantavirus, and discuss the existing knowledge on vaccine and therapeutics against these diseases in order to shed light on the development of new vaccines and treatments.

## The Re-Emergence of Hantavirus

### The Epidemiology of HFRS and HCPS

**FIGURE 1:**
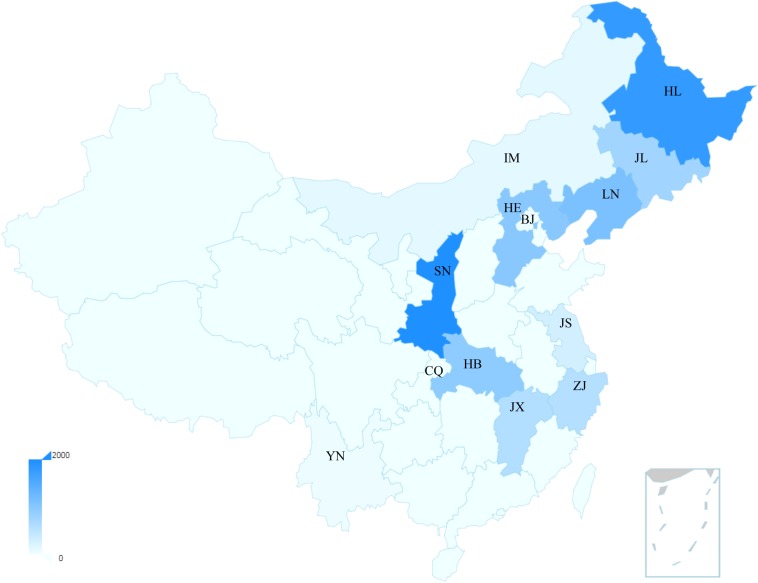
The distribution map of the average hantavirus cases in China of recent years. The main provinces shown in the map were Shaanxi: SN; Heilongjiang: HL; Liaoning: LN; Hebei: HE; Hubei: HB; Jilin: JL; Jiangxi: JX; Zhejiang: ZJ; Jiangsu: JS; Inner Mongoria: IM; Beijing: BJ; Yunnan: YN; Chongqing: CQ. The bar represents the annual average number of cases. These data were published by Chinese literatures.

China has the highest incidence and mortality of HFRS in the world, accounting for more than 90% of the total number of HFRS cases in the world (Zheng et al., 2018). In 2004, the Chinese Center for Disease Control and Prevention (China CDC) established the National Notifiable Disease Surveillance System (NNDSS) online, and HFRS cases of the whole country were reported daily through this system (Zhang et al., 2014). From 2006 to 2012, a total of 77,558 cases and 866 deaths were reported with the average annual incidence rate of 0.83 per 100,000, mortality rate of 0.01 per 100,000 and case fatality rate of 1.13% (Zhang et al., 2014), and its main causative pathogens are HTNV and SEOV (Tian and Stenseth, 2019). So far, HFRS cases have been reported in 30 out of 32 provinces in China (excluding Hong Kong, Macao, and Taiwan) (Zhang et al., 2014). In recent years, the incidence is still high in eastern China (Tian et al., 2017). The distribution map of hantavirus cases reported in recent Chinese literatures is summarized in Figure 1. More than 90% of the total cases were clustered in nine provinces and mainly reported in spring and autumn–winter seasons. We can observe that the annual average number of cases in Shaanxi Province was higher than 2000, ranking at the top of the list (Figure 1). From 2006 to 2017, Shaanxi has gradually become the province with the highest incidence in China, with approximately 4.51 cases/100,000 cases, of which more than 90% are concentrated in the central region (Xi’an, Xianyang, Baoji, and Weinan cities) (Zheng et al., 2018). By November 20 of 2017, 878 people were recorded infected in Shaanxi Province (Dong et al., 2019). We also can observe that the average annual number of cases in the cities of Shannxi, Shandong, and Jiangxi Province rank the top three (Supplementary Figure 1). Another place with a high incidence of HFRS is Qingdao city of Shandong Province, where HFRS incidence is three times higher than the national average, reaching 0.83/100,000 (Jiang et al., 2017).

**FIGURE 2:**
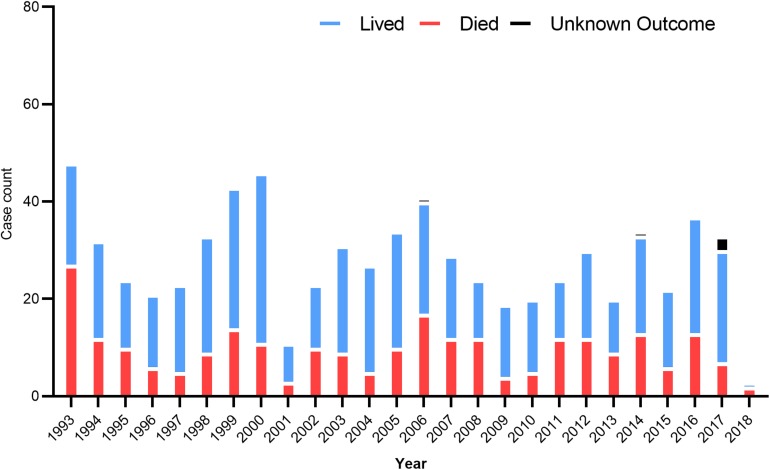
US hantavirus cases from 1993 to 2018. These data were published by the Centers for Disease Control and Prevention, United States.

In the US, 34 states have confirmed and recorded HCPS cases since 1993 (Prince and Lieberman, 2013). In 2008, the first locally acquired case of HFRS caused by the SEOV was confirmed. In 2017, the US CDC investigated an outbreak of SEOV infection that has infected 17 rat owners in seven states (Kerins et al., 2018). The number of hantavirus cases by year in different states is summarized in Figure 2. In Canada, HCPS is still very rare, but cases are recorded every year and show seasonal patterns, mainly between March and May and between September and November. A total of 64 cases have been confirmed since 2000, of which 12 were reported in 2013, most in western Canadian provinces such as Alberta, Manitoba, British Columbia, and Saskatchewan (Kerins et al., 2018). SNV and ANDV are responsible for the majority of hantavirus cases leading to HCPS.

Outside North America, individual cases and small clusters of HCPS have been reported in Balkans, northern Sweden (Bergstedt Oscarsson et al., 2016), Argentina (Pini et al., 2003), Chile (Toro et al., 1998), Poland (Gut et al., 2018), Bolivia (1998), Brazil (Suzuki et al., 2004; Limongi et al., 2009), Serbia (Stanojevic et al., 2019), United Kingdom (Duggan, 2019), Panama (Bayard et al., 2004), and Germany (Ettinger et al., 2012). Overall, in Europe, the incidence of hantavirus infections has steadily increased in recent years: In 2014, a record number of 3754 infections were registered across Europe (Vaheri et al., 2013). Chile has had an average of 67 cases per year since 1995; the disease occurs mainly in spring and summer. However, between June and October 2011, there was an increase in cases and rodent populations (Toro et al., 1998). In Paraguay, an HCPS case was first found in the Chako region in 1995. A total of 56 cases were reported in 2011, and 18 cases were reported in 2012 (Padula et al., 2007). In Panama, HCPS first appeared in 1999, with an average of 12 patients per year. However, 16 cases were reported in 2012, and 14 cases were confirmed as of 21 August 2013. For the first time since 1997, Uruguay has experienced HCPS cases, with an average of nine cases per year. The first case was recorded in northern Uruguay in 2010. In Poland, the infections are caused by mainly the PUUV and DOBV serotypes. The morbidity is not high; it ranges between 0.02 and 0.14 per 100,000 cases, but some papers suggest that the data concerning Poland is underestimated (Gut et al., 2018), because the number of infections then was higher, and it was most likely the epidemic year. In the following year, 2015, there were 6 infections, and in 2016 and 2017, there were 8 and 14 cases registered, respectively. In Germany, from 2001 to 2010, the incidence increased from 0.09 to 2.47/100,000 (Ettinger et al., 2012). In United Kingdom, the virus was first identified in laboratory rats in Scotland in 1977, and all but 1 of the 15 cases of SEOV caused acute kidney injury, which were diagnosed by the Rare and Imported Pathogens Laboratory (Duggan, 2019).

### Hantavirus Virology

**FIGURE 3:**
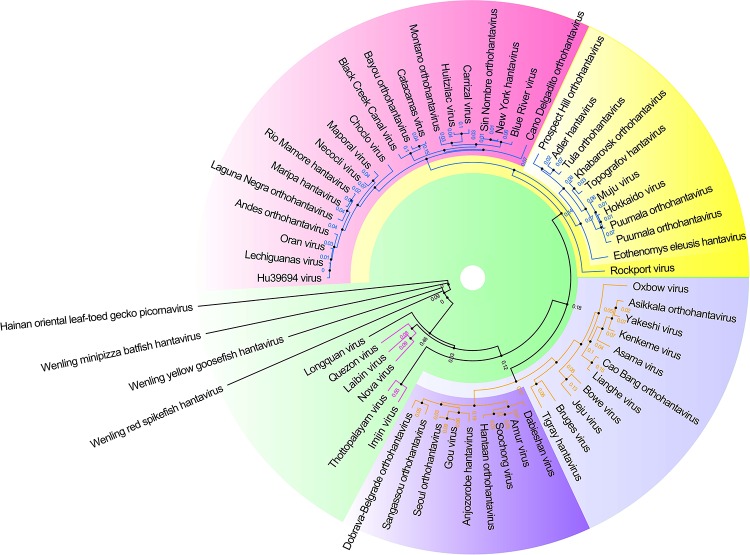
Hantavirus phylogenetic tree on the basis of the M segment sequences. A maximum clade credibility tree of the complete amino acid sequence of the protein encoded by the M segment of viruses belonging to Hantavirus. Different colors represent different clade. These proteins had a high similar signature domain and highly unconserved terminal sequences, which could artificially create similarities between sequences if the alignment was not properly made. Therefore, the phylogenetic tree was made with more robust methods using T-Coffee (default parameter, removed the unconserved sites by filtering the column scores < 4) for multiple sequence alignment and SMS-PhyML (default parameter, bootstrap = 1000, best model = LG +G) for ML (Maximum Likelihood) tree construction.

Hantaviruses belong to the Family *Hantaviridae* of *Bunyavirales* and are a kind of enveloped single negative chain RNA virus (Abudurexiti et al., 2019). A maximum likelihood phylogenetic tree of the complete amino acid and CDS sequence of the M segment of hantaviruses was made based on the international Committee on Taxonomy of Viruses (ICTV) updated taxonomy of the order *Bunyavirales* in 2019 (see Figure 3). This phylogeny shows the modest genetic diversity of the virus family.

The diameter of hantavirus particles is 80–US210 nm, and the structure is spherical or ovoid. They are composed of 20–30% fat, >50% protein, 7% carbohydrates, and 2% RNA. They are very stable and can survive for more than 18 days at 4°C and −20°C and 10 days at room temperature (Vaheri et al., 2013). The genome comprises three negative sense, single-stranded RNAs that consist the small (S), medium (M), and large (L) segments that encode the nucleoprotein (Np), envelope glycoproteins (Gn and Gc), and viral RNA-dependent RNA polymerase (RdRp), respectively (Graham et al., 2019). The outer membrane of hantavirus is composed of Gn and Gc glycoprotein, which mediates the recognition of and entry into host cells. The crystal structure of HTNV Gn is very similar to that of PUUV Gn, which confirms that hantavirus Gn is conserved in hantavirus (Li et al., 2016; Rissanen et al., 2017).

## Clinical Evaluation of Existing Vaccines

Although there have been substantial vaccines, there is no licensed vaccine against hantavirus infection that can be widely used. Despite inactivated hantavirus vaccines being licensed for human use in China and Korea, no such vaccine has been approved in the US or Europe (Tian and Stenseth, 2019). Current clinical studies of inactivated hantavirus vaccine in China or Korea and clinical trials of DNA vaccines in the US are summarized in Table 1.

**TABLE 1:** Existing vaccines in clinical trials and case–control studies.

Number	Title	Conditions	Interventions	Type	Funder Type	Age	Number Enrolled	Date	Status	References
AFMC-17082- IRB-17-077	Effectiveness of inactivated hantavirus vaccine on the disease severity of hemorrhagic fever with renal syndrome	HFRS	Hantavax^®^	Case–control study	Korea	20–22	129	March-09 March-17	Completed	Jung et al., 2018
	Protective effectiveness of inactivated hantavirus vaccine against hemorrhagic fever with renal syndrome	HFRS	Hantavax^®^	Case–control study	Korea	20–30	100	January-11 February-17	Completed	Yi et al., 2018
	Persistence of immune responses to vaccine against hemorrhagic fever with renal syndrome	HFRS	Inactivated hantavirus vaccine	Phase 4	China	16–60	143	June-11 June -13	Completed	Zheng et al., 2018
	The assessment of Hantaan virus-specific antibody responses after the immunization program for hemorrhagic fever with renal syndrome in northwest China	HFRS	HFRS inactivated vaccine [a mixture of HTNV (type I) and SEOV (type II)]	Case–control study	China	16–60	100		Completed	Li et al., 2017
NCT01502345	Study to evaluate the safety, tolerability, and immunogenicity of Hantaan and Puumala virus DNA vaccines	HFRS	HTNV/PUUV DNA vaccine/device combination	Phase 1	U. S. Fed | Industry	18–49	31	January-12 January-13	Completed	Hooper et al., 2014
NCT02116205	Phase 2a immunogenicity study of Hantaan/Puumala virus DNA vaccine for prevention of hemorrhagic fever	HFRS	HTNV/PUUV DNA vaccine	Phase 2a	V. S. Fed | Industry	18–49	132	May-14 July-19	Active, not recruiting	
NCT03682107	Andes virus DNA vaccine for the prevention of hantavirus pulmonary syndrome using the PharmaJetStratis(R) needle-free injection delivery device	HPS	Andes virus DNA vaccine	Phase 1	NIH	18–49	48	February-19 October-19	Recruiting	
NCT03718130	Combination HTNV and PUUV DNA vaccine	HFRS	HTNV vaccine PUUV vaccine HTNV/PUUV virus vaccines	Phase 1	U.S. Fed	18–49	72	May-19 May-21	Not yet recruiting	

### Protective Efficacy of Inactivated Hantavirus Vaccines

The inactivated vaccines comprise entire virions that are inactivated physically (heat) or chemically. In Korea, Lee and An (Cho et al., 2002) first developed an inactivated HTNV vaccine (IHV), which was prepared from the HTNV strain ROK 84/105, which proliferates in the brains of lactating mice. It has been proven that it can induce protein immunization in mice and humans (Yamanishi et al., 1988). In 1990, the Korean HFRS vaccine Hantavax was put into commercial production. The total number of HFRS patients hospitalized in South Korea fell sharply from 1234 in 1991 to 415 in 1997 (Cho et al., 2002). To evaluate the immunogenicity and safety of Hantavax^TM^ in healthy adults in multicenter phase III clinical trials, three dose schedules at 0, 1, and 13 months were used. The seroconversion rate was 90.14% by IFA but only 23.24% by PRNT50 after two primary doses. One month after vaccination, the positive rate of serum was 87.32 and 45.07% according to IFA and PRNT50, respectively. The neutralizing antibody response of the two initial doses of Hantavax^TM^ was very poor. Therefore, it is necessary to carry out enhanced immunization within 2–6 months to provide timely protection for high-risk groups (Song et al., 2016).

From January 2011 to February 2017, the South Korean military conducted a case–control study of 100 patients to evaluate the effect of an IHV on HFRS. The vaccine effectiveness (VE) value of the IHV was 59.1%, but the VE value of HFRS high-incidence area was higher (78.7%) (Jung et al., 2018). However, in 2018, the efficacy of iHV on the progression of HFRS did not show a statistically significant protective effect. From 2009 to 2017, 18 patients inoculated with HFRS vaccine and 110 patients not vaccinated with the HFRS vaccine were recruited at Korean Army Hospital to investigate the severity (AKI) and the efficacy of dialysis events in acute renal injury. Overall, 33.3% of the effective vaccination group had three stages of AKI, compared with 54.5% for the non-vaccinated group. The curative effect of IHV on disease progression was 58.1%, but the curative effect of IHV on HFRS progress did not show a statistically significant protective effect (Yi et al., 2018).

In China, bivalent inactivated vaccines against HTNV and SEOV infection were produced in 1994 and approved by the Pharmacopoeia of China in 2005. Since 2008, the Chinese government has implemented an expanded immunization program targeting HFRS. China uses approximately 2 million doses of HFRS vaccine every year (Schmaljohn, 2012). HFRS cases have significantly dropped to less than 20,000 per year. Phase 4 clinical trials of inactivated hantavirus vaccine showed that the median OD values of IgG antibody were 0.005 (0.004–0.016), 0.116 (0.036–0.620), 0.320 (0.065–0.848), and 0.128 (0.011–0.649), and that the positivity rate was 7.7, 40.6, 62.2, and 48.2% at pre-vaccination, 1 month after the two primary doses, at the booster dose and at 18 months after the booster dose, respectively. Although two main doses can help healthy individuals develop immune responses, the three-dose series should be better than the two-dose series (Zheng et al., 2018). Another clinical study in Xian Yang city in northwest China showed that the positive rate of neutralizing antibody in the unvaccinated group was 10.0%, and the positive rate was 80.0, 90.0, 50.0, and 90%, respectively, at 1, 3, 29, and 33 months after immunization with a vaccine consisting of a mixture of inactivated HTNV and SEOV. This finding indicates that the vaccination program can induce effective humoral immunity in northwestern China and can be maintained up to 33 months after vaccination (Li et al., 2017) (Table 1).

### Clinical Trials of DNA Vaccines for HFRS

At present, DNA vaccines are the most popular method in the research of HFRS and HCPS vaccines, mainly focusing on the use of a hetero-expression system to produce recombinant M protein. DNA vaccines are characterized by safety because they have replication defects, cannot restore the virulence, and cannot spread from person to person or to the environment. A variety of DNA vaccines against the hantavirus envelope glycoprotein gene were developed by Hopper’s group (Schmaljohn et al., 2014). Their studies have confirmed that these DNA vaccines produce neutralizing antibodies in multiple experimental animal species and protected hamsters from HFRS (Schmaljohn et al., 2014).

Next, they developed HFRS candidate DNA vaccines expressing HTNV or PUUV Gn and GC genes and evaluated them in an open-labeled single-center phase 1 study. The results showed that HTNV and PUUV DNA vaccines prepared by electroporation were safe. When mixed together, the response to PUUV was greater than that to the HTNV DNA vaccine, and both DNA vaccines had immunogenicity (Hooper et al., 2014).

The vaccine entered phase 2a trial in 2014 to compare the immune responses to two different doses, 1.0 and 2.0 mg, and mixed HTNV and PUUV DNA vaccines in healthy participants. All groups also received booster doses 6 months after the first vaccination to determine which doses and vaccination plans will be the best way to advance the vaccine development process. To evaluate the safety, responsiveness and immunogenicity of an ANDV DNA vaccine to prevent HPS, the first phase I clinical trials of the ANDV DNA vaccine began in February 2019^1^ (Table 1).

## Pre-Clinical Development of New Vaccines

### DNA Vaccines

Similarly, all DNA vaccines developed against hantavirus target the M gene expressing the envelope GP (Gn and Gc) of hantaviruses (Table 2, part 1).

**TABLE 2:** Pre-clinical development of new vaccines.

DNA vaccine					
Virus	Antigens	Vector	Delivery Methodology	Animal Model	References
SEOV	M/S	pWRG7077	I.M.	Hamsters	Hooper et al., 1999
PUUV	Npeptide	HuAC/pUC19	I.M.	BALB/c mice	Bucht et al., 2001
HTNV and SEOV	M	pWRG/SEO-M,pWRG/HTN-M	I.M.	Rhesus monkeys	Hooper et al., 2001
ANDV	M	pWRG/AND-M	Gene gun	Rhesus macaques	Custer et al., 2003
HTNV and ANDV	M	pWRG/HA-M	Gene gun	Rhesus macaques	Hooper et al., 2006
ANDV	M	pWRG/AND-M	Twin Injector electroporation device. I.M.	Hamster; rabbits	Hooper et al., 2007
HTNV and PUUV	M	pWRG7077	Gene Gun or I.M. EP	Hamsters	Spik et al., 2008
PUUV	M	pWRG/PUU-M	Gene gun	Hamsters	Brocato et al., 2012
ANDV	M	pWRG/AND-M	I.M.	Hamsters; ducks	Brocato et al., 2012
SNV ANDV and SNV HTNV and PUUV Pan-hantavirus	M	pWRG/SN-M	Muscle electroporation (me)	Female New Zealand white rabbits (*Oryctolagus cuniculus*)	Hooper et al., 2013
SNV ANDV	M	pWRG/SN-M, pWRG/AND-M	DSJI system, IM and ID needle-free devices	Rabbits and NHPs (Syrian hamsters; Rhesus macaques; Cynomolgus macaques)	Kwilas et al., 2014
ANDV and SNV	Gp	pWRG/AND-M;pWRG/SN-M	Needle-free jet injection device	Syrian hamsters	Hooper et al., 2014
ANDV	M	pWRG/AND-M	I.M.	Syrian hamsters	Haese et al., 2015
SEOV, HTNV and PUUV	25 Gp epitopes	pcDNA3.1-SHP pGEX-6p-1-SHP	I.M.	BALB/c mice	Zhao et al., 2012
HTNV	Gn	pVAX-LAMP/Gn	I.M.	BALB/c mice	Jiang et al., 2015
HTNV	Gn	pVAX-Gn, pVAX-LAMP and pVAX-LAMP/Gn;	I.M.	BALB/c mice	Jiang et al., 2017

**Subunit Vaccine**					
**Virus**	**Antigens**	**Vector**	**Expression system**	**Results**	**References**

PUUV, TOPV, AMDV and DOBV	NP	pQE-32	*Escherichia coli* cells	Immunization with NP from PUUV, TOPV, ANDV, or DOBV has cross-protection against PUUV	de Carvalho Nicacio et al., 2002
PUUV	NP, G1and G2	pBSK.K27S pWRG7079 K27 G1 pELVS K27 G2 pSC11T7	CV-1 cells COS-7 cells	These recombinant vaccinia viruses were used to detect and clone PUUV-specific CTLs from the PBMC of NE patients. An HLA-A24-restricted CTL line recognizing the G2 protein was isolated and its 9-mer epitope was determined.	Terajima et al., 2002
PUUV, DOBV, and HTNV	NP	pFX7-His6	Yeast *Saccharomyces cerevisiae*	The yield for all nucleocapsid proteins ranged from 0.5 to 1.5 mg per g wet weight of yeast cells	Razanskiene et al., 2004

**Subunit Vaccine**					
**Virus**	**Antigens**	**Vector**	**Expression system**	**Results**	**References**

DOBV	NP	pFX7-derived expression plasmids	Yeast *S. cerevisiae*	The antibodies induced by DOBV rN protein were highly cross-reactive to the rN proteins of HTNV and PUUV. In both mice strains, DOBV rN protein induced N-specific antibodies of all IgG sub classes, suggesting a mixed Th1/Th2 immune response.	Geldmacher et al., 2004
PUUV	Gn and Gc	pTrcHis2 Topo^®^ TA vector	COS-1 cells	The diversity between different Puumala virus: N-linked glycosylation occurs at three sites in Gn (N142, N357, and N409), and at one site in Gc (N937). Also, one possible *O*-glycosylation site was identified in Gc (T985).	Johansson et al., 2004
SEOV	NP	pSTBLUE-1/SEOV-N pFX7-His-SEOV-N	Yeast *S. cerevisiae*	The immunization of a rabbit with the recombinant NP resulted in the induction of a high-titered antibody response and was able to detect antibodies in sera of experimentally infected laboratory rats and in human anti-hantavirus-positive sera or serum pools of patients from different geographical origin.	Schmidt et al., 2005
PUUV	NP	pFD3	*S. cerevisiae* FH4C/pFD3-h-N–GFP	The maximal volumetric yield of N protein was 316 mg L^–1^, the respective yield of h-N protein was 284 mg L^–1^	Antoniukas et al., 2006
PUUV	NP	pTEXmp18	*E. coli* mutant ICONE 200	P40-Puu118 in particular is a good candidate for a recombinant vaccine against PUUV. All recombinant proteins linked to rP40 induced high antibody responses, indicating that rP40 is a carrier protein with potential for use in other vaccines.	Maes et al., 2008

**VLP vaccine**					
**Virus**	**Antigens**	**Vector**	**Expression system**	**Results**	**References**

HTNV	M	pFastBacTM Dual vector	Sf9 insect cells	Chimeric HTNV VLPs containing GPI-anchored GM-CSF or CD40L induced stronger humoral immune responses and cellular immune responses compared to the HTNV VLPs and Chinese commercial inactivated hantavirus vaccine.	Cheng et al., 2016
HTNV	M	pCI-neo	dhfr-deficient CHO cells	*In vitro* stimulation with CD40L or GM-CSF anchored HTNV VLP showed enhanced activation of macrophages and DCs. *In vivo*, it can induce higher level of HTNV specific antibody and neutralizing antibody in mice. Immunized mice splenocytes showed higher ability of secreting IFN-γ and IL-2, as well as enhancing CTL activity.	Ying et al., 2016
HTNV	M	pCI-neo	dhfr-deficient CHO cells	GM-CSF and CD40L VLPs provided stable, long-term protection with a high titer of neutralizing antibody in mice 6 months after immunization. Furthermore, VLPs increased HTNV-specific cellular immune responses via higher expression of IFN-g and CTL responses. HTNV challenge assay results showed long-term protection against HFRS. No significant pathological alteration was observed in the organs of mice after immunization.	Dong et al., 2019

In the United States, a variety of DNA vaccines express the envelope glycoprotein gene of hantaviruses that were developed by Hopper’s group (Schmaljohn et al., 2014). In 1999, HFRS candidate naked DNA vaccine was constructed by the subcloning method. The subcloned cDNA represented the medium fragment (M; encoding G 1 and G 2 glycoprotein) or small fragment (S; encoding nucleocapsid protein) of SEOV and was cloned into the expression vector WRG 7077. Syrian hamsters were vaccinated with the M or S vaccine with a gene gun, and hantavirus-specific antibodies were found in 5 of 5 hamsters or 4 of 5 hamsters, respectively. Evidence of infection was monitored after challenge with SEOV. Twenty-eight days later, hamsters vaccinated with M were protected hamsters from infection, but those inoculated with S were not protected (Hooper et al., 1999).

Then, HTNV M gene products G 1 and G 2 were expressed in 2001, and non-human primates were evaluated. The HTNV M gene has protective effects against HTNV, SEOV, and DOBV in hamsters. The HTNV and ANDV M genes can induce high level of neutralizing antibodies in rhesus monkeys (Hooper et al., 2001). Next, a DNA vaccine plasmid containing the full-length M genome fragment of ANDV (pWRG/AND-M) was constructed (Hooper et al., 2008). Rhesus monkeys inoculated with pWRG/AND-M with gene guns produced very high levels of neutralizing antibodies that neutralized not only ANDV but also other HCPS-related hantavirus strains, such as SNV. On the 4th or 5th day after injection, monkey serum protected 100% of hamsters from fatal diseases (Custer et al., 2003).

Then the pWRG/HA-M plasmid containing the HTNV and ANDV M gene fragments was constructed. Rhesus monkeys were immunized with the pWRG/HA-M vaccine to produce antibodies binding to M gene products (G 1 and G 2 glycoproteins) and neutralize HTNV and ANDV. Subsequently, 1–2 years after the initial vaccination series, the neutralizing antibody titers induced by the double immunogen pWRG/HA-M or a single immunogen expressing only HTNV or ANDV Gp increased rapidly to a high level. This result is the first time that the hantavirus M gene DNA vaccine has been shown to elicit a strong memory response and stimulate an antibody response to neutralize HFRS and HCPS viruses (Hooper et al., 2006; Kwilas et al., 2014) detected the high-titer neutralizing antibody induced by an SNV/ANDV DNA vaccine encoding viral envelope Gp in laboratory animals and non-human primates (NHCPS). It can be delivered effectively using a disposable syringe injection (DSJI) system (Kwilas et al., 2014). Brocato et al. (2013) cloned codon-optimized PUUV M fragments into DNA vaccine vectors to produce the plasmid pWRG/PUU-M, which can produce high-titer neutralizing antibodies in hamsters and NHCPs. The pWRG/PUU-M vaccine protects hamsters from PUUV infection and is not affected by DOBV infection. Unexpectedly, vaccination could protect hamsters in the absence of ANDV cross-neutralizing antibodies in a lethal ANDV disease model (Brocato et al., 2013). Then, the authors tried to produce the pan-hantavirus vaccine with a mixed plasmid DNA vaccine. A study of ANDV hamster model showed that the neutralizing antibody produced by DNA vaccine technology can be used to resist the challenge of an SNV full-length M gene DNA vaccine to prevent the occurrence of HCPS. Rabbits vaccinated with SNV DNA vaccine with muscle electroporation (mEP) produced increased neutralizing antibody titers. In addition, hamsters vaccinated three times with the SNV DNA vaccine with a gene gun were completely free from SNV infection. Rabbits were vaccinated with HCPS mixture (ANDV and SNV plasmid), HFRS mixed (HTNV and PUUV plasmid), or HCPS/HFRS mixture (all four plasmids) by mEP. The results showed that the HCPS mixture and HFRS mixture produce neutralizing antibodies against ANDV/SNV and HTNV/PUUV, respectively. In addition, a mixture of HCPS/HFRS triggered neutralizing antibodies against all four viruses (Hooper et al., 2013). Spik et al. (2008) reported that PUUV and HTNV DNA vaccines should serve as separate regulators. Both vaccines produced neutralizing antibodies when injected alone, but when they were administered as mixtures, only one of the two hantavirus antibodies could be detected. In contrast, if the DNA was administered to an animal as a separate vaccine, a reaction to both was observed. To improve the use of DNA vaccine, a multihead intradermal electroporation device was developed, which can be used to vaccinate with an increased dose of DNA vaccine to the skin. The device will enable multiplasmid vaccine preparation to provide multiplasmid vaccine preparation without interference (Mackow et al., 2014).

In China, a DNA vaccine targeting hantavirus Gn merges the antigen with lysosome-associated membrane protein 1 (LAMP 1), thereby changing the antigen presentation pathway and activating CD4 T cells. The LAMP 1 targeting strategy successfully enhanced the effectiveness of the HTNV Gn vaccine. Further studies showed that pVAX-LAMP/Gn established a memory response during long-term protection (6 months) in mice. Zhao et al. constructed a multi-epitope chimeric DNA vaccine—named the SHP chimeric gene, which contains 25 glycoprotein epitopes of SEOV, HTNV, and PUUV. The humoral and cellular responses were significantly enhanced in vaccinated BALB/c mice (Zhao et al., 2012).

### Subunit Vaccines

Subunit protein vaccines not only are safe and easy to produce but also do not easily cause interference between the components of a multivalent formulation (Sun et al., 2017; Liang et al., 2018; Qu et al., 2018). Because the N protein of hantavirus circulating in different continents can provide high cross-protection in animals, the N protein is considered to be an important part of a wide range of reactive vaccines against hantavirus infection (de Carvalho Nicacio et al., 2002). Research has shown that recombinant nucleocapsid protein (rN) from PUUV, TOPV, ANDV, and DOBV could induce a cross-protective immune response to PUUV. The cross-reaction to PUUV antigen was the highest in the serum of animals immunized with ANDV rN, followed by that to TOPV and DOBV rN. In a proliferation test, T lymphocytes immunized with heterogenic rNs were effectively recalled by PUUV rN *in vitro*, as were animal T lymphocytes immunized with homologous proteins (de Carvalho Nicacio et al., 2002). In addition, recombinant vaccinia virus carrying PUUV N or the first half of the G2 gene was constructed. Detection and cloning of PUUV-specific CTLs from PBMCs of patients with NE by recombinant PUUV vaccinia virus led to the isolation of an HLA-A24-restricted CTL cell line recognizing the G2 protein, and its 9-mer epitope was determined (Terajima et al., 2002). Razanskiene et al. (2004) reported that the stability and high purity of NP of PUUV, DOBV, and HTNV ranged from 0.5 to 1.5 mg/g wet weight of yeast cells. DOBV rN protein is a promising vaccine candidate protein that can induce N-specific antibodies, and its terminal titer is as high as 1:1,000,000 in C57BL/6 mice. The antibody induced by DOBV rN protein has a high cross-reaction with rN protein of PUUV and HTNV (Geldmacher et al., 2004). It was found that there might be RNA fragment exchange between the two PUUV strains. N-linked glycosylation occurred at one site of Gn (N 142, N357, and N409) and GC (N 937), and a possible *O*-glycosylation site was identified in GC (T 985). The study of coding gene products is of great significance for the design of new vaccines (Johansson et al., 2004). Schmidt et al. (2005) reports that NP of SEOV reacts with SEOV-specific monoclonal antibodies and some HTNV- and PUUV cross-reactive monoclonal antibodies. Rabbits immunized with recombinant NP can induce a high-titer antibody response, and NP-specific antibodies were detected in the serum of experimental infected rats and human exposed to hantavirus from different geographical sources. Yeast-expressed SEOV N protein represents a promising antigen for serological epidemiological research and vaccine development (Schmidt et al., 2005). Antoniukas et al. (2006) reported that the biomass and the expression level of recombinant PUUV NP were increased by adding plant extract to YNB medium. The maximum volume yield of the N protein was 316 mg L^–1^ (Antoniukas et al., 2006) (Table 2, part 2).

### VLP Vaccines

Virus-like particle vaccines are similar to natural virus particles but lack infectious genetic material. They are composed of repetitive viral structural proteins with inherent self-assembly characteristics (Yong et al., 2019). How to improve the immunogenicity of VLPs is very important. It was reported that either CD40L- or GM-CSF-contained HTNV VLP expression in sf9 insect cells (Cheng et al., 2016) or CHO cells (Ying et al., 2016) could enhance the activation of macrophages and dendritic cells. CD40L/GM-CSF incorporation into VLPs induced elevated levels of HTNV-specific antibodies and neutralizing antibodies in mice. The spleen cells of immunized mice had an enhanced ability to secrete IFN-γ and IL-2, increasing CTL activity (Cheng et al., 2016). The GM-CSF and CD40L-containing VLPs expressed in a eukaryotic expression vector had a stable, long-term protective effect, with a high titer of neutralizing antibody, on mice within 6 months after immunization (Dong et al., 2019). These results suggest that CD40L/GM-CSF-containing VLPs can be used as a potential candidate vaccine (Table 2, part 3).

## Therapeutic Strategies Against Hantavirus Infection

Hantaviruses primarily infect host capillary endothelial cells of various organs, especially those of kidneys and lungs, and could spark robust immune response in humans. The basic pathological feature of both old world hantaviruses causing HFRS and new world hantaviruses leading to HCPS is dramatically increased vascular permeability, the pathogenesis of which is highly involved in viral infection and excessive immune responses of the host. Extensive capillary leakage results in multiple clinical manifestations, such as hypotensive shock in HFRS and non-cardiogenic pulmonary edema in HCPS, which might deteriorate into multisystem organ failure. Currently, there are no approved post-exposure therapeutic countermeasures against hantaviral infection, but diversified treatment strategies, which target the viral life cycle, host immunological factors or patient clinical symptoms, have been developed and applied to manage HFRS or HCPS (see Table 3). Virus-targeting antivirals, including classical antiviral drugs, antibodies, or novel small molecules, are tested mainly to block hantavirus entry or restrain virus replication. Although several antivirals have been proven to be protective *in vitro* or *in vivo*, there still exist some problems for their clinical application. Host-targeting medicines are designed to improve vascular function or rebuild immune homeostasis, while their curative effects are still under debate. Supportive care is universally applied for HFRS or HCPS, and the specific treatments depend on clinical findings of different disease phases.

**TABLE 3:** Potential therapeutic strategies for hantaviral infection.

Purpose	Drugs	Type	Known/Putative target	Virus/Evidence	Diseases	References
Blocking Viral Entry	MAb Fab 4G2 and 1C9	Human MAbs Fab fragments	Gc glycoprotein	PUUV/Vero E6 cells	HFRS	de Carvalho Nicacio et al., 2000
	Anti-SR rat serum	Rat PAbs	Viral GP	SEOV/newborn rats		Zhang et al., 1989
	3D8, 3G1, 8G3, 8F8, 8G2	Mice MAbs	Gc, or both NP and Gc	HTNV/Vero E6 cells/suckling mice/phase II clinical trials		Xu et al., 2002, 2009
	Patient-derived or vaccine-induced NAbs	Human PAbs	Viral GP	ANDV/hamsters	HCPS	Manigold and Vial, 2014
	Human immune plasma	Human PAbs	Viral GP	ANDV/clinical trials		Vial et al., 2015
	IgY/IgYΔFc	Goose PAbs	Viral GP	ANDV/hamsters		Haese et al., 2015
	JL16 and MIB22	Human MAbs	Viral GP	ANDV/hamsters		Garrido et al., 2018
	Lactoferin	Lactoferin	Viral GP/monocyte and NK	SEOV/Vero E6 cell/suckling mice	HFRS	Murphy et al., 2000, 2001
	Domain III and stem peptides	Peptides	Gc glycoprotein	ANDV, PUUV/Vero E6 cells	HCPS and HFRS	Barriga et al., 2016
	CLVRNLAWC and CQATTARNC	Cyclic nonapeptides	Host receptor	SNV, ANDV/Vero E6 cells	HCPS	Hall et al., 2008
	012-0652, C481-1256 and G319-0078	Peptidomimetic compounds	Host receptor	SNV, ANDV, HTNV/Vero E6 cells	HCPS and HFRS	Hall et al., 2010
	Favipiravir	Pyrazine derivative	RdRp	SNV, ANDV/Vero E6 cells/hamsters	HCPS	Safronetz et al., 2013
Inhibiting Viral Replication	Ribavirin	Nucleoside analogs/mutagen/T cell	RdRp	HTNV, PUUV, ANDV/Vero E6 cells/suckling mice (HTNV)/hamsters (ANDV)	HCPS and HFRS	Mertz et al., 2004; Safronetz et al., 2011; Kobayashi et al., 2012; Chung et al., 2013; Ogg et al., 2013; Westover et al., 2016; Malinin and Platonov, 2017
	ETAR	Nucleoside analogs	RdRp	HTNV, ANDV/Vero E6 cells/suckling mice (HTNV)	HCPS and HFRS	Chung et al., 2008
	K31, K34 and 103772	Small molecules	Viral RNA-NP interaction	SNV, ANDV/Vero E6 cells	HCPS	Salim et al., 2016
	Arbidol	Small molecules	Unclear	HTNV/Vero E6 cells/suckling mice	HFRS	Deng et al., 2009
	siRNA	Small interfering RNA	S, M, and L segments	ANDV/Vero E6 cells	HCPS	Chiang et al., 2014
	siRNA and 3G1-Cκ-tP	Small interfering RNA and Abs	S, M, and L segments	HTNV/Vero E6 cells/suckling mice	HFRS	Yang et al., 2017
Improving Vascular Function	Pazopanib, dasatinib, PP1, bosutinib, and Src inhibitor 1	VEGFR2 kinase inhibitor or SFK inhibitor	Vascular function/VEGF	ANDV/HUVECs	HCPS	Gorbunova et al., 2011
	Vandetanib	Tyrosine-kinase inhibitor	Vascular function/VEGF	ANDV/HUVECs/hamsters	HCPS	Bird et al., 2016
	Ang-1 and S1P	Compounds	Vascular function	HTNV, ANDV, NY-1/HUVECs	HCPS and HFRS	Gavrilovskaya et al., 2008
	Icatibant	Small molecules	BK type 2 receptor	PUUV/clinical case report	HFRS	Antonen et al., 2013; Laine et al., 2015
Rebuilding Immune Homeostasis	Clofilium phosphate	Compounds	Alveolar macrophages	ANDV/hamsters	HCPS	Hammerbeck et al., 2016
	Corticoids or methylprednisolone	Hormone	Immunotherapy	HTNV, ANDV/clinical trials	HCPS and HFRS	Vial et al., 2013; Brocato and Hooper, 2019

### Virus-Targeting Antivirals

#### Blocking Viral Entry

The viral entry process is composed of virus attachment or absorption on susceptible cells, penetration and subsequent uncoating, which is the first step for hantaviral life cycle. Neutralizing antibodies (NAbs) could interact with viral envelope proteins and disturb their binding with receptors on host cells, thus blocking viral attachment. During hantavirus infection, the Gn and Gc glycoproteins, but not nucleocapsid proteins (NP) are considered the major antigens in inducing NAb production (Jiang et al., 2016). High levels of NAbs could be detected in the convalescent phase of HFRS or HCPS patients, and these NAbs could protect the individual from hantaviral infection and have been used as passive immunotherapy (Manigold and Vial, 2014; Jiang et al., 2016). Presently, there are no reported clinical randomized controlled trials using NAbs in immunotherapy for HFRS or HCPS in humans. Most studies measure antibody efficacy through the focus reduction neutralization test (FRNT) and hemagglutination inhibition (HI) test at the cellular level, as well as animal experiments against lethal hantavirus challenge (sucking mice or newborn rat models for HTNV or SEOV infection and hamster models for ANDV or SNV infection) (Zhang et al., 1989; Xu et al., 2002; Manigold and Vial, 2014). For old world hantaviruses, 13 crude Fab preparations directed to the PUUV Gc protein were generated from splenic lymphocytes of a PUUV-immune individual and exhibited type-specific neutralization of PUUV, with a 44–54% reduction in FRNT (de Carvalho Nicacio et al., 2000). Anti-SR-11 (SEOV) rat serum applied 4 h before or 72 h after the challenge could protect against lethal SR-11 (SEOV) infection in newborn rats, and cross-protection effects were also found against KI-262 (SEOV) and 76-118 (HTNV) (Zhang et al., 1989). In China, 18 murine monoclonal antibodies (MAbs) targeting HTNV were prepared in our lab, among which 14 targeted NP, 4 reacted with Gc, and 1 recognized both NP and Gc. The Gc-related MAbs were active in the HI test and displayed high virus-neutralizing activity *in vitro*, and they could be regarded as HTNV NAbs. Consistently, administration of these NAbs within 48 hpi could protect suckling mice from lethal HTNV infection, indicating that NAbs might be a potentially treatment strategy for preexposure prophylaxis and postexposure therapy against HTNV infection (Xu et al., 2002). Phase I clinical pharmacology and toxicity tests for HTNV NAbs in healthy volunteers were performed, and phase II clinical trials to assess the therapeutic efficacy of these NAbs in early stages of HFRS were carried out in endemic settings in China (Xu et al., 2002, 2009). For new world hantaviruses, passive immunization with either patient-derived or vaccine-induced NAbs was capable of protecting against lethal ANDV challenge in hamsters (Manigold and Vial, 2014). A non-randomized multicenter trial using plasma from HCPS convalescent patients for the treatment of acute HCPS has been carried out in Chile, showing that plasma containing high titers of NAbs against ANDV could significantly reduce case fatality rate from 32 to 14% (Vial et al., 2015). Considering the limited availability of convalescent plasma from HCPS survivors, goose polyclonal antibodies were acquired from ANDV DNA vaccine, and the purified IgY/IgY ΔFc from egg yolks could protect hamsters from lethal ANDV infection. Recently, two NAbs, JL16 and MIB22, were developed from the ANDV GP-specific memory B cells of convalescent HCPS patients, both of which reached in a 100% protection rate against lethal ANDV challenge (Garrido et al., 2018). Although NAbs from convalescent patient or immunized animals could obviously restrain hantaviral infection and improve disease outcome, purified human or humanized NAbs against hantaviruses with increased security and efficacy should be developed for clinical experiments. Additionally, research on broadly neutralizing antibodies against hantaviruses is still lacking.

Hantaviral attachment or absorption could also be restrained by lactoferin (LF), an iron-binding glycoprotein that was reported to have broad antibacterial, antifungal, and antiviral activities. LF-pretreated Vero E6 cells showed a reduced number of SR-11 (SEOV) foci, while its antiviral effects were obviously compromised if the LF-pretreated cell monolayer was washed before SEOV infection (Murphy et al., 2000). Further, LF could suppress viral shedding within 24 hpi, the effectiveness of which failed after 48 hpi (Murphy et al., 2001). These results indicated that LF might adhere to cell surface and inhibit SEOV adsorption to host cells. Intriguingly, although LF could not inhibit the expression of NP and Gc, once hantaviral amplification was established in cells, LF contributed to higher survival rates, which might be due to LF-enhanced cytocidal function of natural killer (NK) cells (Murphy et al., 2001). Even so, the specific mechanisms of how LF inhibits SEOV absorption and influences host immune responses, as well as the effects of LF on other species of hantaviruses, remain obscure.

Hantaviral fusion with cell membrane facilitates its entry process. The Hantavirus Gc envelope glycoprotein acts as a viral fusion protein that is essential for viral entry. It has been demonstrated that Gc shares similar features with class II fusion proteins, which means that three domains of viral fusion proteins are connected by a stem region anchoring in the viral envelope (Cifuentes-Munoz et al., 2011). Exogenous protein fragments containing fusion protein domain III (DIII) and the stem region could bind to the core of the fusion protein and interfere with its conformation transition, inhibiting the membrane fusion process. Based on this effect, soluble recombinant peptides that mimic DIII and the stem region were prepared, and were proven to block both ANDV and PUUV infection in Vero E6 cells by blocking membrane fusion (Barriga et al., 2016). The strategy using viral Gc DIII and stem fragments to suppress fusion might be feasible for other hantaviruses, while the protective effects *in vivo* still need further studies to confirm.

Furthermore, antivirals targeting hantaviral receptors have been synthesized. It has been demonstrated that pathogenic hantaviruses attach to the cell surface via host-specific α_IIb_β_3_ orα_v_β_3_integrins while non-pathogenic hantaviruses initiate cellular entry relying on α_v_β_1_ integrins (Jiang et al., 2016). Based on the structure of cyclic peptides known to bind the α_v_β_3_ receptor, a few of cyclic peptides or small molecules were designed and screened for their antihantaviral function. The cyclic nonapeptides CLVRNLAWC and CQATTARNC could inhibit SNV and ANDV infection *in vitro* (Hall et al., 2008). After two rounds of biological screening, the peptidomimetic compounds 8012-0652, C481-1256, and G319-0078 were screened out with potency in the nanomolar range against infection of a panel of hantaviruses, including SNV, ANDV, and HTNV (Hall et al., 2010). Further studies should be performed to evaluate the safety and efficacy of these small molecules *in vivo*.

#### Inhibiting Viral Replication

Viral proteins are the working molecules for viral biosynthesis, among which RdRp plays an important role in hantaviral transcription and replication and is considered a desirable drug target. The pyrazine derivative Favipiravir and the nucleoside analogs Ribavirin (RBV) and ETAR have been tested effective antihantaviral drugs that directly or indirectly affect the biological function of RdRp. Favipiravir (Avigan; T-705) was initially discovered in 2002 as an antiviral drug selectively inhibiting the RdRp of influenza virus and then reported to have a high activity against a panel of Bunyaviruses (Gowen et al., 2007; Westover et al., 2016). Favipiravir could attenuate the viral RNA replication level and decrease the progeny virus yield of SNV and ANDV *in vitro*. *In vivo* studies, including non-lethal SNV challenged and lethal ANDV challenged hamster model, demonstrated that oral administration favipiravir at the dosage of 100 mg/kg twice daily could prominently reduce viral load in hamster serum and various organs and resulted in 100% survival in the ANDV lethal infection model (Safronetz et al., 2013). Delayed favipiravir administration after the onset of viremia exerted no protective effects against ANDV infection. Notably, there is no reported clinical trial with favipiravir as an antiviral treatment in HFRS and HCPS.

RBV and ETAR are nucleoside analogs that interfere with viral replication. They can inhibit inosine monophosphate dehydrogenase and reduce the synthesis GTP *de novo*, hence affecting the function of viral RdRp. As a potent mutagen, RBV could induce RNA mutagenesis in subsequent generations of HTNV virions (Chung et al., 2013), while it is not expected that ETAR induces mutation, probably due to the lack of pseudobase pair presence (Chung et al., 2008). Additionally, RBV was reported to modulate host immune responses by suppressing interleukin-10-producing regulatory T cells (Kobayashi et al., 2012), while there is no evidence showing that ETAR could exert immunoregulatory effects (Szabo, 2017). Both *in vitro* and *in vivo* antihantaviral activity of RBV and ETAR have been confirmed by a series of studies. For HFRS therapy, RBV-treated suckling mice had a higher survival rate upon HTNV infection than the placebo control group (Huggins et al., 1986). In China, a double-blind placebo-controlled clinical trial enrolled 242 HFRS patients caused by HTNV infection. The result showed that postexposure administration of RBV could decrease mortality by sevenfold and reduce the risk of entering the oliguric phase, suggesting that RBV is effective against HTNV-induced HFRS (Huggins et al., 1991). However, a clinical trial for HFRS caused by PUUV infection in Russia showed that RBV could not improve patient condition, especially reducing viral load, and that RBV could increase the occurrence of side effects of RBV, including rash, sinus bradycardia, hyperbilirubinemia, and reduced hemoglobin. These data suggested insufficient efficacy and safety of RBV in the treatment of HFRS caused by PUUV (Malinin and Platonov, 2017). For HCPS treatment, two studies confirmed that RBV could protect hamsters from lethally intraperitoneal or intranasal ANDV challenge without toxicity, and even abbreviated treatment regimens from 7 days to 3 days worked if therapy commenced 1 day following virus challenge (Safronetz et al., 2011; Ogg et al., 2013). Unfortunately, two clinical trials for the treatment of HCPS using RBV did not show any improvement in survival rates compared with those for patients during the same time frame or receiving placebo treatment. It is speculated that RBV treatment might be ineffective once HCPS progresses to the cardiopulmonary phase. ETAR showed an EC(50) value of 10 and 4.4 μmol/L for HTNV and ANDV in Vero E6 cells, respectively, which is much lower than its toxic dosage of 880 μmol/L (Chung et al., 2008). Moreover, ETAR administration in sucking mice with a dosage of 12.5 or 25 mg/kg at 10 days post HTNV infection could significantly increase the survival rate from 10 to 25%, an effect equal to that of RBV (Chung et al., 2008). To date, there were no studies on the antiviral activity of ETAR on other hantaviruses.

Hantaviral NP could bind to an evolutionary conserved sequence at the 5′ terminus of hantaviral genomic RNA. The interaction of NP with the viral genome could protect viral RNA from host recognition and degradation, facilitate the N-mediated viral RNA translation process, and help package the viral genome into nucleocapsids. Several compounds, namely, lead inhibitor K31, K34, and 103772, were reported to interrupt the NP-RNA interaction against SNV and ANDV infection. They could abrogate both viral RNA synthesis and translation without affecting the normal biological process of host cells, among which K31 showed antiviral activity similar to that of RBV (Salim et al., 2016). However, K31 failed to affect the replication of HIV or adenovirus, demonstrating its selectivity for hantaviruses. Arbidol, an immunomodulator developed in Russia, was also found to protect against HTNV infection both *in vivo* and *in vitro* (Deng et al., 2009).

Targeting viral RNAs is the most direct and effective way to curb hantaviral replication. Small interfering RNA (siRNA) directed against hantaviral genes could facilitate viral RNA clearance based on the RNA interfering (RNAi) mechanisms and has been tested as a potential antiviral strategy *in vitro* and *in vivo*. It has been demonstrated that siRNAs targeting the S, M, or L segment of ANDV could reduce viral replication in Vero E6 cells or human lung microvascular endothelial cells and that an S-targeted siRNA pool seemed to be more efficient in reducing viral transcription and replication than M- or L-targeted siRNA in Vero E6 cells. Importantly, these siRNAs could inhibit ANDV replication even if given after infection (Chiang et al., 2014). Although siRNAs could effectively suppress hantavirus amplification in host cells most likely through promoting viral RNA clearance, their antiviral activity might be greatly compromised considering their poor biological stability and targeting ability *in vivo*. One strategy is to combine siRNAs targeting encoding sequences of HTNV genome with recombinant antibodies (3G1-Cκ-tP) recognizing HTNV Gc, which were applied by intraperitoneal injection in an HTNV-induced encephalitis mouse model. The result indicated that through combination, siRNAs could be specifically delivered to the HTNV-infected brain cells and protect against HTNV intracranial infection (Yang et al., 2017). On all accounts, novel delivery system should be developed to ensure the stability and selectivity of siRNAs, and the efficacy and safety of these systems remained unclear for the treatment of HFRS or HCPS.

### Host-Targeting Medicines

#### Improving Vascular Function

Increased capillary leakage due to hantaviral infection is the basic pathogenic feature for both HFRS and HCPS. Therefore, treatment strategies improving microvascular endothelial cell function seem to be feasible in mitigating disease severity and reducing mortality (Alkharsah, 2018). Hantavirus-disturbed vascular function is a multifactorial event whose complicated mechanisms still need to be elucidated, and two kinds of hypothesis have been developed. The vascular endothelial growth factor (VEGF) theory was first proposed and studied in depth. VEGF binding to VEGF receptor 2 (VEGFR2) could activate SFK (Src family kinases) signaling, which may result in dissociation, internalization, and degradation of VE-cadherin. Altered expression and localization of VE-cadherin contributed to impaired barrier structure of adherent junctions, which could lead to incremental cellular permeability (Jiang et al., 2016). It has been demonstrated HTNV or ANDV infection could disrupt the interaction of β3 integrin with VEGFR2 and induce VEGFR2 hyper phosphorylation, which may enhance the permeability of infected endothelial cells by sensitizing them to VEGF (Gavrilovskaya et al., 2012; Wang et al., 2012). As increased VEGF content has been noted in the plasma of HFRS and HCPS patients and is closely related to disease severity in the acute phase (Bird et al., 2016; Pal et al., 2018), it is feasible to repurpose those FDA-approved drugs targeting vasoactive mediators for use as hantaviral infection therapy. In line with this strategy, one study reported that the VEGFR2 kinase inhibitor, as well as SFK inhibitors, could obviously stabilize ANDV-induced endovascular permeability, among which the SFK inhibitors dasatinib and pazopanib blocked VE-cadherin dissociation by more 90% (Gorbunova et al., 2011). Another study also indicated that application of vandetanib, a tyrosine-kinase inhibitor preventing VEGFR2 phosphorylation, before ANDV infection could delay animal lethality and increase total survival by 23% in ANDV-challenged hamsters (Bird et al., 2016). In contrast, similar small molecules administered after the onset of viremia failed to protect hamsters from lethal ANDV challenge (Brocato and Hooper, 2019). Moreover, some other small molecules, such as angiopoietin 1 (Ang-1) and sphingosine 1-phosphate (S1P), were found to inhibit hantavirus-directed endothelial cell permeability *in vitro* (Gavrilovskaya et al., 2008), while further research *in vivo* should be performed to confirm their efficacy.

Another promising theory is increased activation of the kinin–kallikrein system (KKS) during hantavirus infection. One study using a model with co-cultured endothelial and vascular smooth muscle cells demonstrated that activation of KKS and subsequent liberation of bradykinin (BK), but not VEGF, were mainly responsible for the dramatic increase in endothelial cell permeability after hantavirus infection (Taylor et al., 2013). BK, a nonapeptide that binds BK type 2 receptor, could induce blood vessel dilatation and vascular permeability increase, resulting in collapsed blood pressure. Icatibant is a peptidomimetic drug that can block the interaction of BK with the BK type 2 receptor by binding to this receptor itself. One case reported that a 37-year-old male who once underwent splenectomy due to congenital spherocytosis manifested with severe capillary leakage syndrome caused by PUUV infection. With a single dose of icatibant, the condition of the patient stabilized, followed by gradual improvement and full recovery (Antonen et al., 2013). Another case report also confirmed the efficient treatment of icatibant in a 67-year-old female HFRS patient (Laine et al., 2015). These clinical data indicated that BK receptor antagonist might be a novel treatment strategy for hantavirus diseases. Nevertheless, it should also be noted that the foresaid two patients had spleen abnormalities, which might be related to the curative effect of icatibant. Clinical trials enrolling a large number of HFRS or HCPS patients should be performed to further identify the remedy effects of bradykinin receptor antagonists.

#### Rebuilding Immune Homeostasis

It is wildly accepted that HFRS and HCPS are caused by uncontrolled systemic inflammatory responses, in which multiple inflammatory cytokines, especially TNF-α, IL-8, and RANTES, contributed to disease progression (Manigold and Vial, 2014; Schonrich and Raftery, 2017); however, immunoregulation treatment in HFRS or HCPS is undesirable. A recent study with depletion of alveolar macrophages, which are considered the main resource for proinflammatory responses, could not prevent ANDV-caused pathogenesis in hamsters (Hammerbeck et al., 2016). The immunomodulatory treatment of corticoids was firstly performed during the Korean war, but the case fatality rate was not improved (Sayer et al., 1955). In Chile, a retrospective analysis suggested that a high dose of methylprednisolone could reduce mortality in 22 HCPS patients (Brocato and Hooper, 2019). However, a double-blind clinical trial in Chile failed to observe a significantly improved outcome between methylprednisolone recipients and placebo recipients for HCPS. Similarly, another randomized prospective study did not show any benefit from corticosteroid treatment in HFRS patients (Qian et al., 1990).

### Supportive Care

The initiation of careful observation and prompt but judicious supportive treatment is crucial to improve patient survival condition for both HFRS and HCPS (Sargianou et al., 2012). It has been demonstrated that admission to the ICU and supportive treatment could greatly reduce the mortality rate of HFRS (Huggins et al., 1991). In general, the treatment principle for HFRS patients is using intravenous hydration and electrolyte therapy to maintain physiological blood pressure. Platelet transfusions can be applied to reduce the mortality in patients with severe thrombocytopenia. Intermittent hemodialysis (IHD) is the first choice to improve uremia condition and rectify kidney dysfunction in patients with acute kidney injury. Continuous renal replacement therapy (CRRT) should be applied for those critical HFRS patients, especially when they have a complication, such as multi-organ injury pulmonary edema, or cerebropathy (Jiang et al., 2016). Treatment of patients with HCPS should also be performed in the ICU with continuous cardiac monitoring and respiratory support. The palliative treatments for HCPS usually include mechanical ventilation, extracorporeal membrane oxygenation, and hemofiltration (Sargianou et al., 2012).

## Discussion

HFRS and HCPS caused by hantavirus infection are reemerging infectious diseases that greatly threaten global public health. At present, there are currently no US FDA-approved treatments or vaccines available; only the whole virus-inactivated vaccine against HTNV or SEOV is available in China and Korea. With the implementation of intervention measures, the incidence of hantavirus infections seems to have shown a decline in recent years. In China and Korea, the number of HFRS cases has been drastically reduced. But the vaccines elicit suboptimal immune responses, confer inadequate protection, and may cause safety concerns. In 2017, the recurrence of global outbreak of HFRS has drawn renewed attention to this old disease, which seriously threatens human health. HFRS in China was still a natural focal disease with relatively high morbidity and fatality, and its distribution and epidemic trends had also changed. Surveillance measures, together with prevention and control strategies, should be improved and strengthened to reduce HFRS infection in China. Therefore, the best solution is to develop a functional vaccine to prevent hantavirus infection. Among the three types of vaccines discussed above, only DNA vaccine candidates have progressed to clinical trials. Subunit protein vaccines not only are safe and easy to produce but also do not easily cause interference between the components of a multivalent formulation (Sun et al., 2017; Liang et al., 2018; Qu et al., 2018). To resolve the above problems, we propose to construct a universal genetic engineering novel subunit protein vaccine against HTNV and SEOV by combining bioinformatics methods, viral surface protein structure biology knowledge, and molecular biology tools.

Antiviral treatment only works when applied during the early infection stage, possibly because uncontrolled immune responses occur and predominate the pathogenesis process post-acute infection. Immunomodulatory therapy hardly improves the patient survival rate, possibly because suppressed inflammatory responses inhibit prompt viral clearance and enhance virus-caused injury increase. Therefore, based on rapid supportive care, effectively combining antiviral treatment and immunomodulatory therapy is a potential strategy for HFRS and HPS. 3G1 and 3D8, the mice MAbs against HTNV developed by our team, have been used for HFRS treatment and the result indicated that application of 3G1 and 3D8 at early stage of disease could significantly improve the patient condition and increase survival rates, especially for those severe or critical HFRS patients (data unpublished). Hence, NAbs might be the most promising treatment for HFRS or HPS, and the effective humanized neutralizing antibodies should be further developed.

Moreover, it is universally acknowledged that type I IFN responses are essential for hosts to defend against hantaviral infection. Multiple IFN stimulated genes (ISG) were confirmed to have antihantaviral activity. The interferon-induced MxA protein, a GTPase with extensive antiviral activity, notably against influenza viruses, was reported to inhibit HTNV and PUUV replication in Vero cells (Frese et al., 1996). The interferon-induced IFITM3 protein was able to inhibit HTNV infection in both HUVEC and A549 cells by inhibiting virus entry (Xu-Yang et al., 2016). Several studies have shown that pretreatment with type I IFN could effectively inhibit hantaviral infection. Pretreating endothelial cells (ECs) with IFNα blocks hantavirus replication, and this inhibitory effect is still observed when IFNα is added to ECs within 12 hpi; however, the addition of IFNα 15–24 h after infection had little effect on hantavirus replication. Clinical data indicated that IFN treatment is only effective prophylactically or shortly after hantavirus infection. In fact, during the natural infection process, compared with non-pathogenic hantaviruses, pathogenic hantaviruses could inhibit host IFN production at an early infection stage, but the specific mechanism remains ambiguous. Our team recently found that HTNV could induce complete autophagy at an early phase, which promotes host MAVS degradation and disturbs RIG-I-MAVS signaling-mediated IFN production. The application of autophagy inhibitors, including 3-MA and CQ, could significantly enhance type I IFN responses and inhibit HTNV replication both *in vitro* and *in vivo*. We also demonstrated that lncRNA NEAT1 could positively regulate RIG-I-DDX60-mediated IFN responses during HTNV infection and that overexpression of NEAT1 could restrain HTNV amplification both *in vitro* and *in vivo*. These results suggest that enhancing host IFN responses during the early infection phase may be a novel therapeutic strategy for HFRS and HCPS, while there is still much work to be done to translate basic medicine research to clinical practice.

## Author Contributions

RL and HM wrote the manuscript. JS, MH, and ZL provided published evidence. QZ made the phylogenetic tree. XJ, FZ, and XW edited, reviewed, and approved its final version.

## Conflict of Interest

The authors declare that the research was conducted in the absence of any commercial or financial relationships that could be construed as a potential conflict of interest.

## Footnotes

1 www.clinicaltrials.gov
