# Supplementary material for: Vaccines and Therapeutics Against Hantaviruses
Source: Front Microbiol. 2020 Jan 30;10:2989. doi: 10.3389/fmicb.2019.02989 (PMC7002362; doi:10.3389/fmicb.2019.02989)
Supplement: Supplementary file 1 [file Image_1.pdf]

Supplementary Material

Supplementary Figures

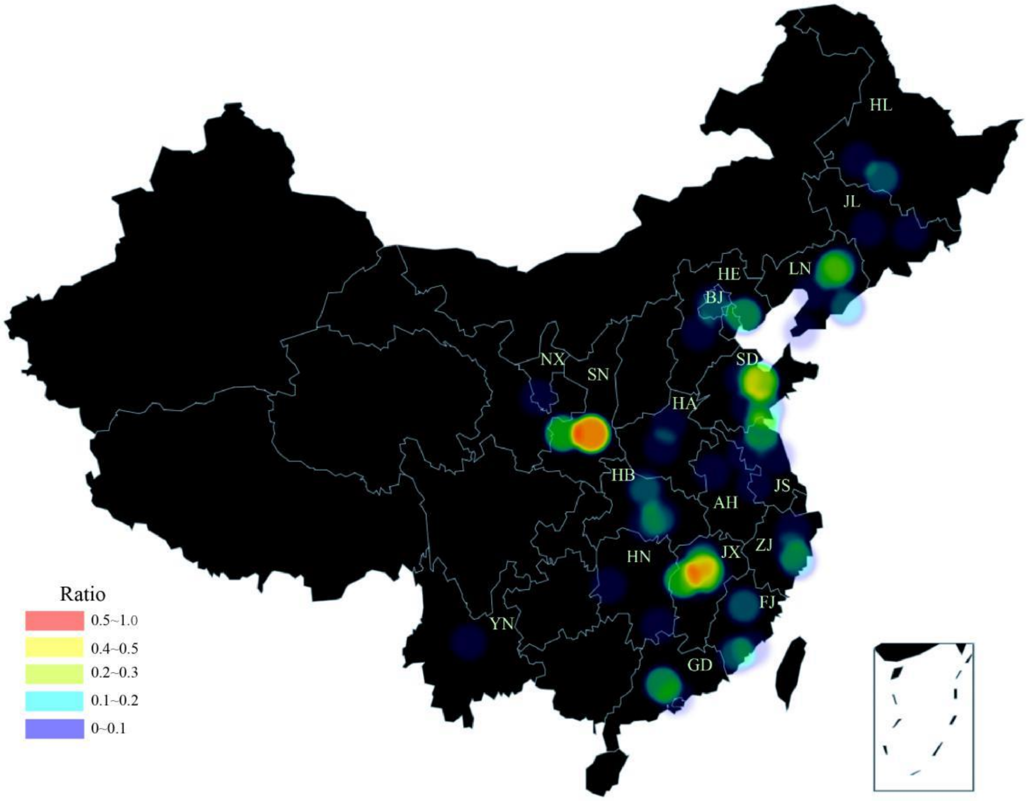

Supplementary Figure 1. The distribution map of the average hantavirus cases in China of recent years. The main cities shown in the map were belong to the following provinces: Beijing: BJ; Fujian: FJ; Guangdong: GD; Hebei: HE; Henan: HA; Heilongjiang: HL; Hubei: HB; Hunan: HN; Jilin: JL; Jiangsu: JS; Jiangxi: JX; Liaoning: LN; Ningxia: NX; Shandong: SD; Shaanxi: SN; Yunnan: YN; Zhejiang: ZJ; Anhui: AH;. The bar represents the ratio normalized using the maximum.

* These data were published by Chinese literatures.
